# Action Monitoring for Equity and Gender in Health

**DOI:** 10.3329/jhpn.v26i3.1902

**Published:** 2008-09

**Authors:** Abbas Bhuiya, S.M.A. Hanifi, Shehrin Shaila Mahmood

**Affiliations:** ICDDR, B, Mohakhali, Dhaka 1212, Bangladesh

**Keywords:** Equity, Gender, Health, Health services, Monitoring tools, Bangladesh

## Abstract

Equity and gender, despite being universal concerns for all health programmes in Bangladesh, are often missing in many of the health agenda. The health programmes fail to address these important dimensions unless these are specifically included in the planning stage of a programme and are continually monitored for progress. This paper presents the situation of equity in health in Bangladesh, innovations in monitoring equity in the use of health services in general and by the poor in particular, and impact of targeted non-health interventions on health outcomes of the poor. It was argued that an equitable use of health services might also result in enhanced overall coverage of the services. The findings show that government services at the upazila level are used by the poor proportionately more than they are in the community, while at the private facilities, the situation is reverse. Commonly-used monitoring tools, at times, are not very useful for the programme managers to know how well they are doing in reaching the poor. Use of benefit-incidence ratio may provide a quick feedback to the health facility managers about their extent of serving the poor. Similarly, Lot Quality Assurance Sampling can be an easy-to-use tool for monitoring coverage at the community level requiring a very small sample size. Although health problems are biomedical phenomena, their solutions may include actions beyond the biomedical framework. Studies have shown that non-health interventions targeted towards the poor improve the use of health services and reduce mortality among children in poor households. The study on equity and health deals with various interlocking issues, and the examples and views presented in this paper intend to introduce their importance in designing and managing health and development programmes.

## INTRODUCTION

Equity and gender are universal concerns for all health programmes in Bangladesh. There will always be groups who have better health and more advanced health services than other groups, but society cannot accept differences which are considered not ‘reasonable’ or inhumane. A child born to a poor family should still receive at least basic health services that are available to wealthier families and are appropriate. Similarly, the health of a female child needs the same level of care and support as a male child, and women need health services that are geared to their needs.

Gender and equity are concepts that are frequently missed unless these are specifically addressed at the planning stage of a programme and are continually monitored for progress. Sometimes, gender issues arise in areas where they are least expected, but when gender-related observations are made, these frequently open new understandings of health and disease. An example is the differential case-fatality rates for women infected with visceral leishmaniasis (1). Women receive treatment for their infection significantly later in the illness, and likely for this reason, their chance of dying from the infection more than doubles. In contrast, rates of tuberculosis are much higher among men than women, again pointing to the underlying causes, most likely social, that if known will help in controlling the disease (2).

## ASSESSING EQUITY AND THE POTENTIAL BENEFIT OF CORRECTING INEQUITY

Unfortunately, many indicators show that inequity persists in health outcomes and health services in our country. The programmes must constantly adjust their efforts to ensure that they are reaching the poor. This paper reviews some recent findings from Bangladesh on equity. There have been many ways to express how equitable health services are, and some of these measures are reviewed here. As a concept, however, one may start by examining what would happen to the ‘average’ health statistics if there were a change in services provided such that services provided to the richest quintile were available to all. This approach compares the indicators if services provided to the rich were available to all. While this can be done for each country, The table shows the results for Bangladesh.

As shown in the table, instead of an average child mortality rate of 24 per 1,000 children aged 1-4 year(s), this would drop to 7. Rather than an average under-five mortality rate of 88 per 1,000 livebirths, this would drop to 72. Rather than having 73% of children fully immunized, this figure would climb to 87%. And the percentage of wasted stunted, and underweight children would drop from 13%, 43%, and 48% to 9%, 25%, and 30% respectively.

**Table:** Child health, immunization, and safer motherhood practices, Bangladesh, 2004

Parameter	Average rate/percentage	Rate/percentage in the highest quintile
Child health		
Child mortality	24	7
Under-five mortality	88	72
Immunization (%)	73.1	86.7
Nutritional status of children		
Wasting (%)	12.8	9.4
Stunting (%)	43	25
Underweight (%)	47.5	30.2
Safe motherhood		
At least 1 antenatal care visit (%)	55.9	84.1
Postnatal care visit (%)	17.8	46.9
Caesarean section (%)	3.5	14.4
Source: Bangladesh Demographic and Health Survey 2004 (3)		

For indicators of safe motherhood, the number of pregnant women who received at least one antenatal visit would increase from 56% to 84%. Similarly, the proportion of postnatal visit would increase from 18% to 47%. For caesarean section, the average rate is 3.5% which goes up to 14% for the wealthier quintile. [The caesarean-section rate must be interpreted with caution since it seems that the caesarean-section rate for the wealthier group is unnecessarily high, and not all procedures were medically indicated.]

## HOW TO IDENTIFY THE POOR

Several tools have been developed to identify the poor. Household income and consumption data are commonly used for measuring poverty. Poverty can also be estimated by determining the number or proportion of individuals whose income or consumption fall below a certain minimum level, known as the poverty-line. An internationally-accepted poverty-line proposed by the World Bank is US$ 1 a day.

The asset quintile has also been widely used in measuring poverty throughout the world. The asset quintile only requires information on ownership of assets of a household. However, the analysis regarding this tool is highly technical, and the scores generated through this measure are not readily interpretable. This method, thus, provides little assistance to field officers in carrying out programme interventions and pro-poor monitoring, the traditional method of measuring poverty using land ownership and occupation criterion is more practical. Additional methods are being validated which take into account the different dimensions of poverty, such as whether the family has food security, education, employment, and skills, and whether the family has connections to assist them in times of trouble.

## PRO-EQUITY MONITORING

Many programmes attempt to be ‘pro-poor’, but these have difficulty in effectively developing the programmes or monitoring how well these address the needs of the poor. Even programmes that attempt to target the poorest of the poor rarely reach this group despite best intentions. National data (e.g. the BDHS) are not helpful to programme managers when monitoring the use of services by the poor. These data are collected very infrequently and do not represent the catchment population of the manager. If a local manager intends to monitor whether the programme is equitable, he/she needs monitoring-tools that can lead to improvements in the programme as needed. The lack of such suitable management tools creates a real constraint for pro-poor programmes, and there is a need for innovations in this field.

The Lot Quality Assurance Sampling methods which require relatively small sample sizes have the potential to provide such a pro-poor monitoring tool, but they need further evaluation to become institutionalized. Among the many advantages of this technique, it can be carried out by local field officers, and its results are immediately obvious to managers, once the basic monitoring methods are understood. Programmes with failing marks can then be adjusted to improve performance, and the monitoring method can be repeated many times as needed to continually upgrade services (4-6). The method has been widely used for monitoring the success of programmes such as EPI, but it can be equally well-adapted for monitoring equity and gender issues in health services (7).

**Fig. 1:**
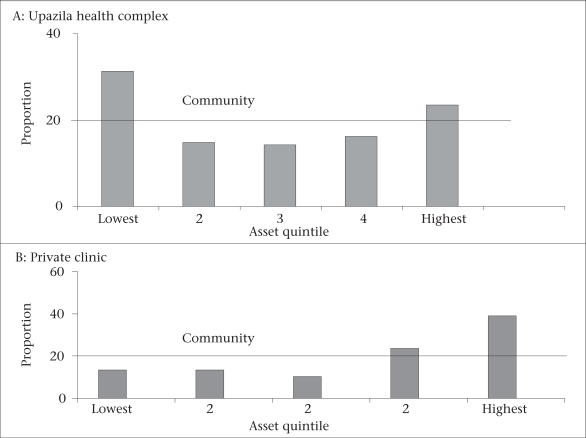
Proportion of patients in each quintile at an upazila health complex and a private clinic

Another technique, which has recently been promoted by the World Bank, is benefit-incidence ratio. This technique basically compares the proportion of poor in the community with those among facility users. If the facility is equitable, one would expect the same proportion of poor people using the facility as found in the community. The findings from an upazila in Bangladesh are illustrated by the charts (Fig. 1), which demonstrate the use of the upazila health complex and a nearby private clinic. It can be seen that more of the lowest quintiles are represented in the upazila health complex than these are in the community. While for the private clinic, it is reverse (7).

## WHY THE POOR DO NOT USE SERVICES

Several studies have sought to understand reasons behind the low use-rate of public and private health services among the poor compared to their rich counterparts (8,9).

- Public-sector services, although officially free, actually are not. Service recipients often have to pay unofficial ‘tips’ for these services
- There is an inadequate supply of medicines at the facility; there is, thus, little incentive to attend the service since the patient still must purchase medicines or supplies
- Quality of care is perceived to be low, and patients are not treated with respect
- Private-sector services are expensive and unaffordable for most poor patients
- There are additional indirect costs, e.g. transportation, referral and lost time, and cost of medicines
- Distance to the facility may be long
- There are cultural barriers—a lack of confidence in the healthcare system, and ignorance about the nature of health conditions

## PRO-EQUITY NATURE OF IMMUNIZATION SERVICES

Different types of health services are not equally equitable, and preventive programmes tend to be much more equitable than curative services. This has been especially true for measles vaccination; however, the same principles are likely to apply to all vaccines. This issue is fairly obvious given the great difference in services accessed by and provided to poor vs richer families. Nearly everyone can receive a vaccine, and this can be obtained at a convenient time; thus, the illness can be prevented. If an illness strikes, it may be that only the rich will avail of treatment. The more acute the situation is, the greater is the difference between the rich and the poor. Thus, time of and access to treatment are both factors in determining who gets treated. An illustration of that is severe pneumonia or severe dehydrating diarrhoea. In each case, if there is delay in receiving proper treatment, the outcome can be fatal, and in both cases, the poor are disadvantaged in receiving treatment quickly.

Successes of the EPI, along with the wide-scale distribution of vitamin A capsules, are likely to be among the major reasons for the past improvements in child-survival rates. However, a potential caution in this observation is that the benefits of the current strategy of EPI and vitamin A may now have been realized, and one should not expect a further downward trend in child mortality unless additional interventions are introduced to further reduce specific causes of death, especially among the poor.

**Fig. 2:**
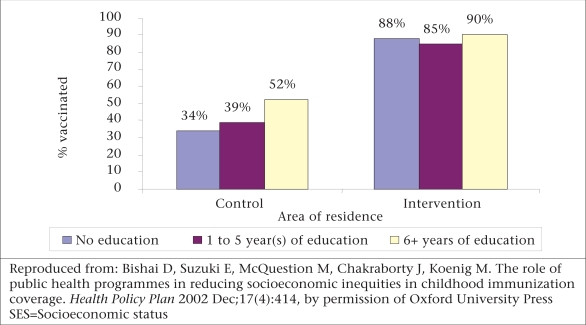
SES gradients in measles vaccination by area of residence

**Fig. 3:**
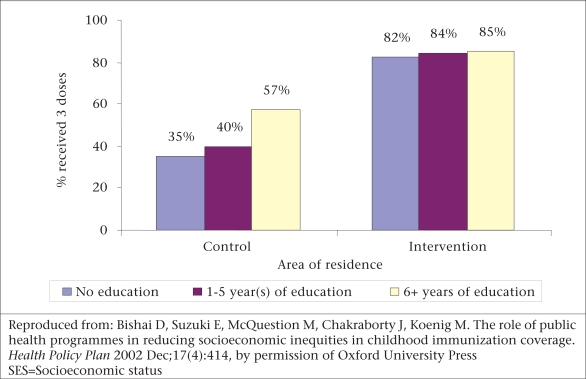
SES gradients in DPT vaccination by area of residence

Results of the study by Bishai suggest that an aggressive provision of public-health programmes, such as the MCH-FP programme in Matlab during the 1980s, played a significant role in narrowing or eliminating prevailing differentials in receipt of vaccines relating to socioeconomic status (10). In this study, the intervention families were actively visited and encouraged to receive the vaccines, whereas the families in the comparison area were receiving vaccines through a ‘routine programme’. The important finding was that the active intervention overcame the barrier pertaining to socioeconomic status in delivering this health service. The graphs (Fig. 2 and 3) show that the vaccination coverage was higher in the intervention area, but especially striking is the gradient of vaccination status by mother's schooling which appeared in the comparison area but not in the intervention area.

## NON-HEALTH INTERVENTIONS

### Impact of a development programme

Equity concerns must also take into account that some interventions, which are not directly related to heath, may still have an important health outcome if they help improve equity. An example of this is the study showing the impact of a woman-focused development programme on child survival in Matlab, Bangladesh. In this intervention, the programme focused on the formation of women's groups for saving and credit, training on skills development, functional literacy, including legal and social awareness, and technical and marketing support to projects undertaken with the loan money from the organization. The unexpected result of the intervention was a reduction in infant mortality among children in the group of women who participated in the programme.

**Fig. 4:**
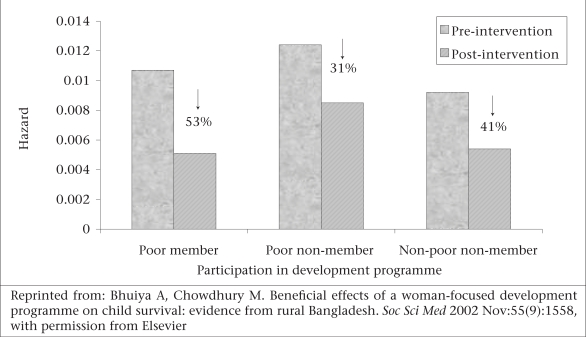
Predicted hazards of infant deaths during pre- and post-intervention periods by participation of mothers in a development programme, Matlab, 1988–1997

It is evident from Figure 4 that the decline in the risk of death over time during infancy was the largest (53%) for children of mothers who joined the development programme, followed by children of rich (41%) and poor non-members (31%). The difference between the gains among children of mothers who are members of the programme and that of poor non-members (22%) may be attributed to the beneficial effects of the development programme (11).

The topic of equity and health is a vast one, and this paper has only begun to skim the surface. However, it is hoped that these examples will introduce their importance in developing health and development programmes.
